# Evolutionary history of endemic Sulawesi squirrels constructed from UCEs and mitogenomes sequenced from museum specimens

**DOI:** 10.1186/s12862-016-0650-z

**Published:** 2016-04-14

**Authors:** Melissa T. R. Hawkins, Jennifer A. Leonard, Kristofer M. Helgen, Molly M. McDonough, Larry L. Rockwood, Jesus E. Maldonado

**Affiliations:** Center for Conservation and Evolutionary Genetics, Smithsonian Conservation Biology Institute, National Zoological Park, Washington, DC 20008 USA; Division of Mammals, National Museum of Natural History, MRC 108, Smithsonian Institution, P.O. Box 37012, Washington, DC 20013-7012 USA; Department of Environmental Science and Policy, George Mason University, Fairfax, VA 22030 USA; Conservation and Evolutionary Genetics Group, Estación Biológica de Doñana(EBD-CSIC), 41092 Sevilla, Spain; Department of Biology, George Mason University, Fairfax, VA 22030 USA

**Keywords:** Wallacea, Ultraconserved elements, Species tree, Sciuridae, Ancient introgression

## Abstract

**Background:**

The Indonesian island of Sulawesi has a complex geological history. It is composed of several landmasses that have arrived at a near modern configuration only in the past few million years. It is the largest island in the biodiversity hotspot of Wallacea—an area demarcated by the biogeographic breaks between Wallace’s and Lydekker’s lines. The mammal fauna of Sulawesi is transitional between Asian and Australian faunas. Sulawesi’s three genera of squirrels, all endemic (subfamily Nannosciurinae: *Hyosciurus*, *Rubrisciurus* and *Prosciurillus*), are of Asian origin and have evolved a variety of phenotypes that allow a range of ecological niche specializations. Here we present a molecular phylogeny of this radiation using data from museum specimens. High throughput sequencing technology was used to generate whole mitochondrial genomes and a panel of nuclear ultraconserved elements providing a large genome-wide dataset for inferring phylogenetic relationships.

**Results:**

Our analysis confirmed monophyly of the Sulawesi taxa with deep divergences between the three endemic genera, which predate the amalgamation of the current island of Sulawesi. This suggests lineages may have evolved in allopatry after crossing Wallace’s line. Nuclear and mitochondrial analyses were largely congruent and well supported, except for the placement of *Prosciurillus murinus*. Mitochondrial analysis revealed paraphyly for *Prosciurillus*, with *P. murinus* between or outside of *Hyosciurus* and *Rubrisciurus*, separate from other species of *Prosciurillus.* A deep but monophyletic history for the four included species of *Prosciurillus* was recovered with the nuclear data.

**Conclusions:**

The divergence of the Sulawesi squirrels from their closest relatives dated to ~9.7–12.5 million years ago (MYA), pushing back the age estimate of this ancient adaptive radiation prior to the formation of the current conformation of Sulawesi. Generic level diversification took place around 9.7 MYA, opening the possibility that the genera represent allopatric lineages that evolved in isolation in an ancient proto-Sulawesian archipelago. We propose that incongruence between phylogenies based on nuclear and mitochondrial sequences may have resulted from biogeographic discordance, when two allopatric lineages come into secondary contact, with complete replacement of the mitochondria in one species.

**Electronic supplementary material:**

The online version of this article (doi:10.1186/s12862-016-0650-z) contains supplementary material, which is available to authorized users.

## Background

The Indonesian island of Sulawesi has a complex geological history. The current ‘k’ shaped landmass consists of four peninsulas and a central core [1]. Geologists have estimated the movement and extent for each fragment of the island complex [2, 3], and determined that the present conformation is relatively recent (limited to the past ~5 million years). The four major peninsulas correspond to landmasses from at least three tectonic provinces, and two smaller continental fragments [4, 5]. Throughout geologic history, Sulawesi has dramatically changed in configuration, area, and elevation [2, 6, 7]. The first appearance of what we presently consider Sulawesi was the western peninsula, or west Sulawesi, during Eocene sea level changes (~40 MYA) [8]. In the mid Oligocene (~30 MYA) west Sulawesi was surrounded by ocean, but terrestrial habitats were present. At this time, east Java and south Sulawesi were still submerged in shallow marine environments (as determined by carbonate deposits) [8]. Rapid geologic changes ~23 MYA (in the Cenozoic) resulted in the collision of the Australian plate (specifically the Sula spur) and the Sunda Shelf, which led to some degree of mountain formation in Sulawesi and modified the elevation of eastern Sulawesi. The southern peninsula continued to be at least partially submerged at this time, and west Sulawesi was relatively flat. The nature of the connection between eastern and western Sulawesi is unknown. The geological record is scarce for this period of time, but the few poorly dated marine sediments available indicate that the area may have been largely subaerial [8].

During the mid Miocene a process known as ‘rollback’ was likely ongoing near the subduction zone around the north peninsula of Sulawesi [8]. Essentially this process involves the expansion of terrestrial environments surrounding a subduction zone when the inflow of heated mantle pushes to the surface, elevates, and expands the terrestrial environment in the opposite direction of the plate movement (see Figures 11 and 12 in [8]). This rollback process likely caused a long-lived terrestrial environment in Sulawesi [8]. Additional evidence for extended terrestrial habitat comes from central and southeastern Sulawesi, where a significant amount of erosion and prolonged weathering has been documented. Much of Sulawesi was exposed land during the Miocene, but few additional details are available regarding the extent of land and elevation through time [5, 8].

The present topography of Sulawesi dates to the Late Miocene, approximately 5 MYA, when tectonic movements affected the amount of exposed land and extent of mountains across Sulawesi [8]. Although sea level changes during the Pleistocene had an extreme effect on the exposed land area and therefore mobility of species across the Sunda Shelf, the effect on Sulawesi was minor [5, 9–12]. The combined isolation and steep edges of tectonic shelves on Sulawesi likely prevented a terrestrial connection between islands in Wallacea [8]. The complex geology of this biodiverse island has confounded many studies trying to determine the age of endemic lineages, which is where phylogenetic reconstruction can aid in understanding [5, 13].

Mammals on Sulawesi are primarily of Asian descent, except for the phalangerid marsupials and pteropodine bats, which have Australian or Pacific origin [14, 15]. The earliest estimated mammalian colonization of Sulawesi was by phalangerid marsupials approximately 21–23 MYA [14]. Squirrels are estimated as the second oldest arrivals, around 11 MYA [16]. Subsequent mammalian colonization events (all of which occurred from Asia, and have been dated) occurred during the Pliocene (2.6–5.3 MYA) and include the first colonization events for both macaques and shrews [17–19]. Interestingly, macaques and shrews both colonized Sulawesi a second time in the Pleistocene (0.01–2.6 MYA). Other mammalian colonization events occurred primarily during the Pleistocene, including bovids and suids [20–24], although recent re-dating recovered vastly different estimates with a trend towards much older colonization events [5]. The estimated date of arrival of the squirrels, however, remained largely unchanged.

**Fig. 1:**
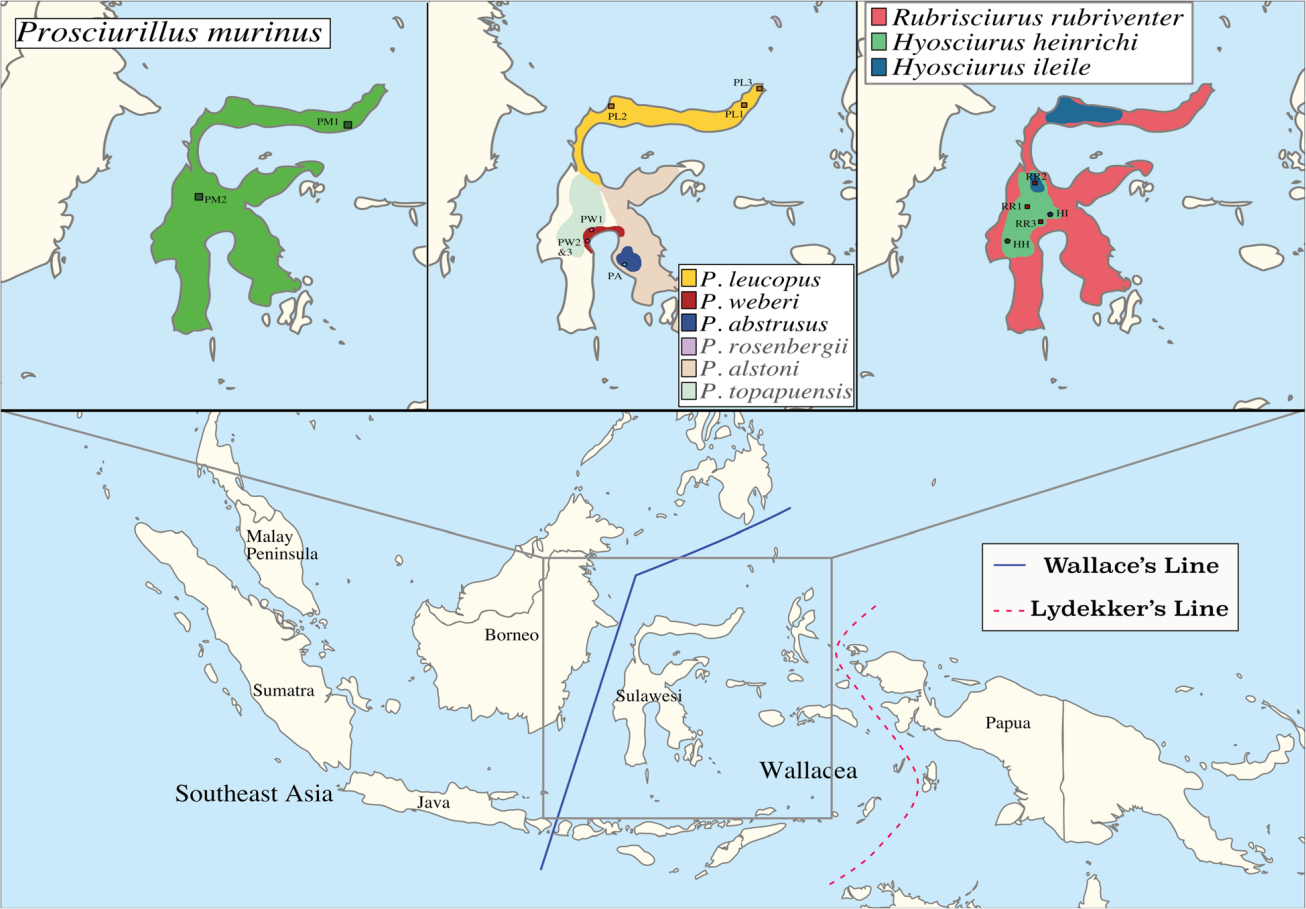
Map of Sulawesi with biogeographic barriers shown, as well as the approximate distribution of each species plotted within the inlaid maps. Species distributions estimated from [27, 28]. Individual samples are placed in the same color, and on the representative map of each sampled species. Species not included are in grey text in the center inlaid map, and *P. rosenbergii* is distributed on the islands north of the northern peninsula (Sangihe Islands). Base maps modified from Wikimedia Commons

With the exception of a recent morphological and ecological analysis that detailed the distribution and characterization of each species [25], squirrels are some of the least studied taxa on Sulawesi. Three genera of endemic squirrels (subfamily Nannosciurinae/Callosciurinae, referred to hereafter as Nannosciurinae) occur on Sulawesi: *Hyosciurus*, the long-nosed squirrels; *Rubrisciurus*, the giant ground squirrels; and *Prosciurillus*, the relatively small-bodied tree squirrels. The genus *Hyosciurus* contains two named species, *H. ileile* and *H. heinrichi*. These terrestrial species have extremely elongate rostra, possibly for a specialized invertebrate diet [26]. Both species of *Hyosciurus* have fairly restricted ranges, and *H. ileile* has two disjunct recorded populations, one in the northern peninsula and the second in the central core, although the species is probably more widespread [25, 26]. *Hyosciurus heinrichi* is distributed through the highlands of the central core. The genus *Rubrisciurus* contains a single species, *R. rubriventer*, which is distributed across the entire island. Finally, *Prosciurillus* is the most diverse genus, containing seven recognized species (*P. abstrusus, P. alstoni, P. leucomus, P. murinus, P. weberi, P. topapuensis,* and *P. rosenbergii*- located north of Sulawesi, on four small islands), all of which (except *P. murinus*) have restricted ranges (Fig. 1). Several *Prosciurillus* species are found in specific habitat types, for example, *P. weberi* occurs in mangrove forests, and *P. topapuensis* and *P. abstrusus* in montane forests [25, 27]. The three genera are easily distinguished morphologically. *Hyosciurus* has a very long rostrum, *Rubrisciurus* is very large and bright red, while the species of *Prosciurillus*, the most diverse and variable genus, are brownish or olivaceous in general coloration. Members of this latter genus usually have ear tufts of various colors and banding along the tail, and some have either patches of coloration along the nape (*P. leucomus*) or dorsal stripes (*P. weberi*) [25, 26, 28]. The smallest and most plain squirrel, *Prosciurillus murinus*, lacks ear tufts and tail banding.

To date, only one sciurid-wide phylogeny has included three of these species, one representative per genus (*Prosciurillus murinus, Rubrisciurus rubriventer* and *Hyosciurus heinrichi*) [16]. Mercer and Roth [16] analyzed two mitochondrial genes (12S and 16S rRNA) and one nuclear gene (IRBP), based on which they suggested that a single squirrel lineage crossed Wallace’s Line to give rise to these three genera of squirrels on Sulawesi. Here we estimated the phylogeny of endemic Sulawesi squirrels using relatively dense taxonomic sampling across Sulawesi and several Sundaland outgroup taxa in order to test the hypothesis that there was a single colonization event for Sulawesi squirrels. We compared divergence time estimates to what is known of the geological history of Sulawesi in order to determine which geological events may have been important in the history of the group. We sequenced whole mitochondrial genomes (mitogenomes hereafter), which provide much greater resolution than single mitochondrial genes for evaluating phylogenetic relationships, especially over short time scales [29, 30]. Here we analyze all non-saturated coding genes of the sequenced mitogenomes. Given that mitochondrial DNA is inherited as a single unit, species tree estimates require additional independently inherited markers [31]. Therefore, we enriched for a panel of nuclear markers, ultraconserved elements (UCEs), to derive sequences from the variable flanking regions [32, 33], which have proven useful for resolving rapid radiations in a variety of taxa [32, 34]. Fresh tissue samples for most of the species are not available; therefore, we obtained our molecular data from historic museum skins, mostly collected approximately a century ago. Since rates of nucleotide substitution are greater in flanking regions of UCEs and degradation has an effect on DNA fragment length, this study provides empirical evidence for the utility of UCEs in degraded DNA studies. We also report the enrichment success and the assembled length for the recovered UCEs to build our phylogenies.

## Results

### Mitogenomes

Whole mitochondrial genomes were successfully constructed from 14 individuals from seven of the 10 recognized species (Table 1). Average mitogenome coverage was 232.2× and ranged from 46.5 to 880.8× (Table 2).

**Table 1:** All samples included in this study, with museum catalog #, geographic location, Genbank # (for mitogenomes), whether mtDNA or UCEs were extracted for each sample, and the number of UCEs enriched

Species	Catalog #	Location	Genbank accession #	mtDNA	UCEs	# Loci
1 *Hyosciurus heinrichi*	MZB 34908	Sulawesi	KR911797	x		258
2 *Hyosciurus ileile*	ANMH 225461	Gunung Kanino, Sulawesi	KR911796	x	x	2608
3 *Prosciurillus abstrusus*	AMNH 101360	Menkoka Mts., Tanke Salokko	KR911793	x	x	3748
4 *Prosciurillus leucomus occidentalis 1*	AMNH 196571	Roeroekan, Sulawesi	KR911786	x	x	3248
5 *Prosciurillus leucomus leucomus 2*	USNM 200274	Sulawesi, Toli Toli	KR911785	x	x	1262
6 *Prosciurillus leucomus leucomus 3*	USNM 216771	Sulawesi, Teteamoel	KR911784	x		
7 *Prosciurillus murinus 1*	USNM 217817	Sulawesi, Temboan	KR911795	x	x	1067
8 *Prosciurillus murinus 2*	USNM 218713	Sulawesi, Koelawi	KR911794	x		
9 *Prosciurillus weberi 1*	MZB 6252	Mosamba, Celebes	KR911789	x		
10 *Prosciurillus weberi 2*	MZB 6254	Mohari, Sulawesi	KR911787	x		
11 *Prosciurillus weberi 3*	MZB 6255	N. Celebes, Meuado	KR911788	x		
12 *Rubrisciurus rubriventer 1*	USNM 218710	Sulawesi, Koelawi	KR911791	x	x	1359
13 *Rubrisciurus rubriventer 2*	USNM 218711	Sulawesi, Rano Lindoe	KR911790	x		
14 *Rubrisciurus rubriventer 3*	ANMH 101313	Mengkoka Mts., Tanke Salokko, Sulawesi	KR911792	x		
15 *Exilisciurus exilis*	ROM 102254	East Kalimantan, Indonesia	KR911801	x	x	3706
16 *Callosciurus adamsi*	NZP95-322	Sabah, Malaysia	KR911800	x	x	3834
17 *Nannosciurus melanotis*	USNM 123098	Sumatra, Indonesia	KT001463	x	x	3800
18 *Lariscus insignis*	MVZ192194	Sumatra, Indonesia	KR911799	x	x	3838
19 *Sundasciurus everetti*	MTRB 3/16	Mount Kinabalu, Sabah, Malaysia	KR911798	x	x	3863
20 *Callosciurus erythraeus*	KM502568	China	KM502568	x		

Museum abbreviations: MZB; Museum Zoologicum Bogoriense, Bogor, Java, Indonesia; AMNH; American Museum of Natural History, New York, New York, USA; USNM; National Museum of Natural History, Smithsonian Institution, Washington D.C, USA; NZP; National Zoological Park, Washington D.C., USA; MTRB; M.Hawkins Field Samples, see Hawkins et al. [41] for details

**Table 2:** Results of mitochondrial genome enrichment for Sulawesi samples (modern outgroup samples were generated from Long Range PCR), read merging (PEAR), and read mapping (with BWA); average coverage of mitogenome also detailed

	Catalog #	Species	Location	# merged reads	# reads mapped w/Sulawesi reference	Average coverage
1	MZB 34908	*Hyosciurus heinrichi*	Sulawesi	745249	80370	775.8
2	ANMH 225461	*Hyosciurus ileile*	Gunung Kanino, Sulawesi	428430	76952	367.4
3	AMNH 101360	*Prosciurillus abstrusus*	Menkoka Mts., Tanke Salokko	147485	35982	152.7
4	AMNH 196571	*Prosciurillus leucomus occidentalis 1*	Roeroekan, Sulawesi	348234	69856	513.3
5	USNM 216771	*Prosciurillus leucomus leucomus 3*	Sulawesi, Teteamoel	140615	35892	111.5
6	USNM 200274	*Prosciurillus leucomus leucomus 2*	Sulawesi, Toli Toli	123089	21723	64.9
7	USNM 218713	*Prosciurillus murinus 2*	Sulawesi, Koelawi	145631	36348	105.6
8	USNM 217817	*Prosciurillus murinus 1*	Sulawesi, Temboan	113550	25655	75.3
9	MZB 6254	*Prosciurillus weberi 2*	Mohari, Sulawesi	9706968	27230	174.7
10	MZB 6255	*Prosciurillus weberi 3*	N. Celebes, Meuado	2296646	166994	1082.6
11	MZB 6252	*Prosciurillus weberi 1*	Mosamba, Celebes	177965	9705	59.9
12	ANMH 101313	*Rubrisciurus rubriventer 3*	Mengkoka Mts., Tanke Salokko, Sulawesi	106941	12385	66.4
13	USNM 218711	*Rubrisciurus rubriventer 2*	Sulawesi, Rano Lindoe	70224	24618	178.1
14	USNM 218710	*Rubrisciurus rubriventer 1*	Sulawesi, Koelawi	25633	19029	84.2

Evidence of contamination was detected in two *Prosciurillus murinus* samples (MZB 5973 and 5977), and one sample of *Prosciurillus weberi* (MZB 6256) had low coverage (16X); these were therefore excluded from phylogenomic analysis. Contamination was deduced based on damage which occurred during transport of samples (tube seals were damaged for both samples MZB 5973 and 5977), which was later combined with the recovery of a large number of herterozygosities within these two specimens. In order to assure that submitted sequences were of the species intended; these samples were removed from further analysis. All samples were checked for contamination by repeating library preparation, replicate sequencing, translation of coding genes, and by evaluating heterozygous sites in the mitochondrion. Nuclear copies of mitochondrial sequences have been shown to be an issue when using historic material as a genetic source [35]. In order to ensure that our sequences represented mitochondrial sequences as opposed to nuclear copies, we followed the standard protocols for high throughput sequencing (HTS hereafter) generated mitogenomes and determined the mitochondrial sequence by consensus of high coverage fragments (as in [36]).

**Fig. 2:**
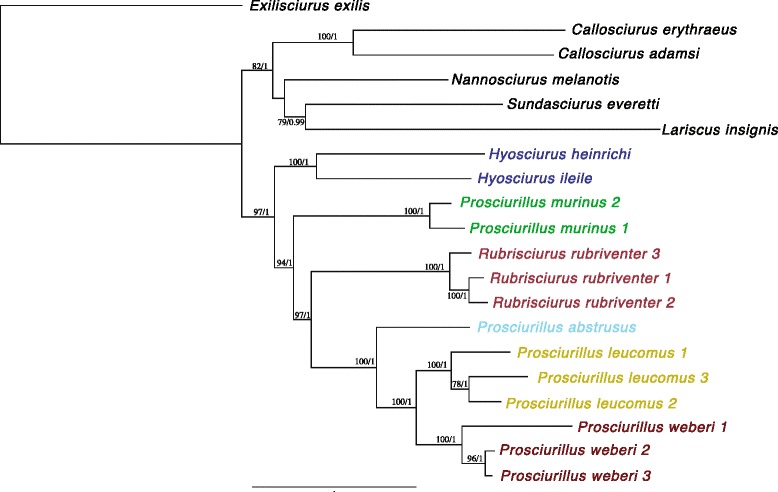
Whole mitochondrial genome phylogenetic tree. Support values are provided for both Maximum Likelihood and Bayesian Inference (ML/BI). Colors correspond to range maps in Fig. 1. Species with multiple individuals are detailed in Table 1

**Fig. 3:**
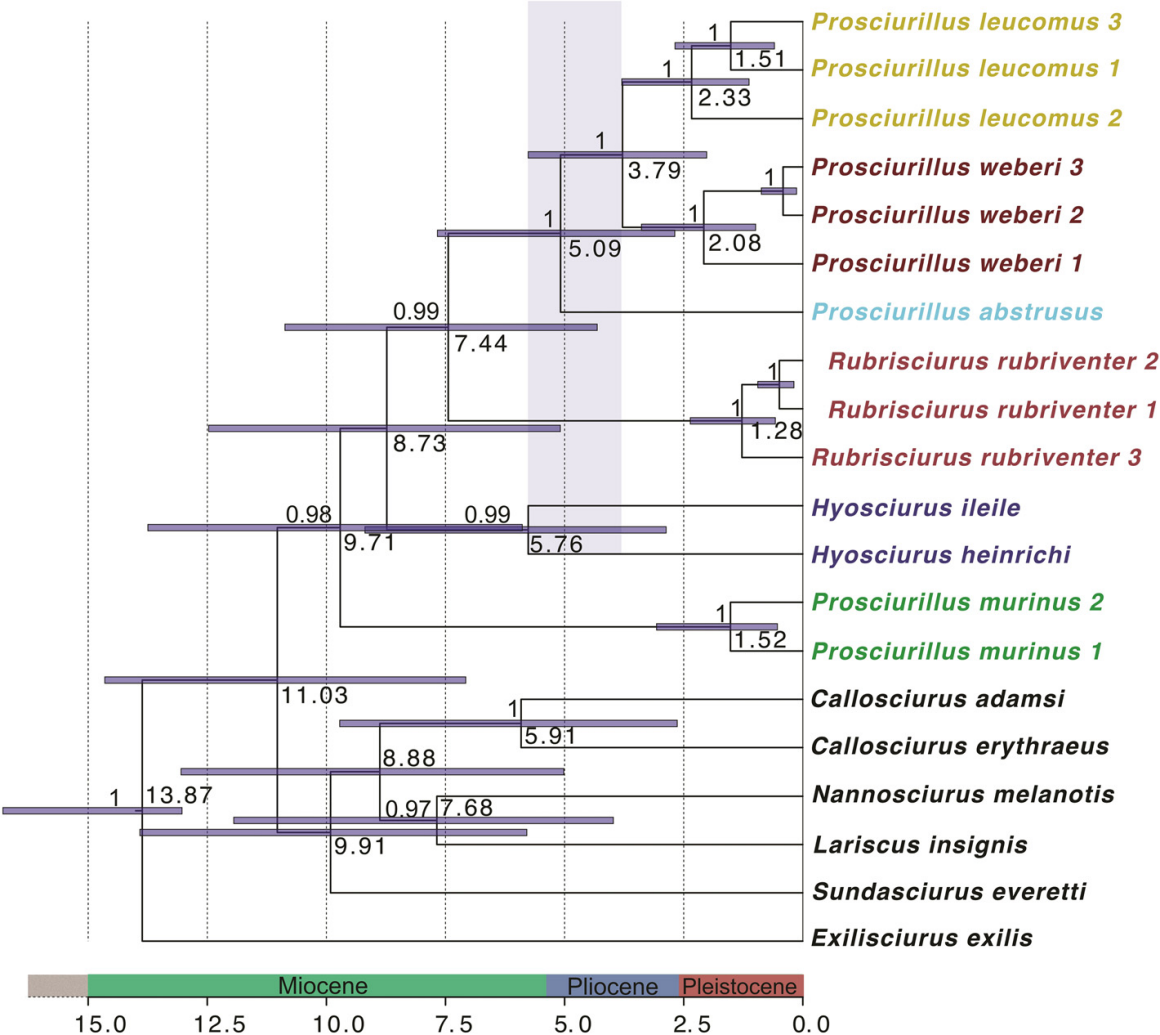
Divergence dated whole mitochondrial genome phylogeny. Bayesian inference support values are shown and dates are provided in millions of years. *Blue bars* indicate the 95 % HPD for each dated node. The split from Sundaland to Sulawesi taxa is the node dated to 11.03 MYA

Several mitochondrial genes were found to have near-saturated third codon positions; therefore, only non-saturated genes (cytochrome oxidase subunit 1–3, and ND1-5) coding genes were included in phylogenetic inference. Phylogenetic trees generated from the whole mitochondrial genome, without including gene or codon partitioning, were used for comparison to the topology when data partitions were applied (data not shown, complete mitogenomes are available on GenBank, accession numbers presented in Table 1). Both maximum likelihood (ML) and Bayesian inference (BI) estimates supported the monophyly of Sulawesi squirrels (Fig. 2; BS ≥75 and BPP ≥ 0.95). Ingroup nodes were well-supported (BS >90 and BPP >0.98) and the topology was consistent with Mercer and Roth [16], indicating that the genus *Hyosciurus* was the earliest diverging lineage of Sulawesi squirrels. Interestingly, *Prosciurillus* was found to be paraphyletic, with *Prosciurillus murinus* falling out sister to *Rubrisciurus* + *Prosciurillus* (Figs. 2 and 3). Divergence dating replicates implemented in BEAST [37] were found to converge and had high ESS values (>200). The empty alignment was significantly different from the mitogenome alignment and recovered poor ESS values, indicating the priors did not strongly influence the results. Coalescent modeling recovered a slightly different topology than the BI and ML analyses, with *Prosciurillus murinus* the most divergent species within the radiation of Sulawesi squirrels, followed by the *Hyosciurus*, *Rubrisciurus*, and finally the remaining *Prosciurillus* species (Fig. 3). All Sulawesi squirrels shared a common ancestor almost 10 MYA (HPDs = 5.89 – 13.75). Intraspecific divergence times varied between taxa (1.5–2.3 MYA). Interestingly, the two *Hyosciurus* species were deeply divergent, sharing a common ancestor ~5.8 MYA (Fig. 3).

### UCEs

UCE loci were successfully sequenced (range 1035 to 3748; Table 1) for all but one Sulawesi species (258 recovered UCE loci, *Hyosciurus heinrichi* MZB 34908); therefore, this sample was excluded from subsequent analyses. Outgroup specimens from high quality tissues samples yielded more loci (3706 to 3863 loci; Table 1) than degraded DNA samples. Twenty-eight loci were successfully characterized in all taxa (total length 8824 bps). The incomplete matrix contained 4046 loci (1,743,442 bps), in which 2646 loci contained at least one parsimony informative site per locus. The average length of loci in the full dataset was 434 bps, ranging from 150 (minimum length acceptable) to 2863 bps. This dataset was reduced to include only loci with at least three informative sites (1137 loci) and then for alignments that contained at least 9 of the 11 included taxa (362 loci). This partition of the dataset averaged 435 bps, and ranged from 192–918 bps. This resulted in a concatenated alignment of 157,353 bps. In order to see if the length and compositions of various loci had an effect on topology, an incomplete matrix containing UCEs with at least 8 of 11 taxa was generated, consisting of 2410 loci (956,549 bps long). To mirror the complete dataset of 28 loci, another subset of the 28 ‘most informative’ loci was constructed, which contained the 28 loci with the highest number of parsimony informative sites (13,578 bps in length). A comparison of the complete dataset with the ‘most informative’ loci recovered overall mean genetic distance of 0.005 % in the complete dataset, and from 0.030 % in the ‘most informative’ dataset (Additional file 1: Table S1).

**Fig. 4:**
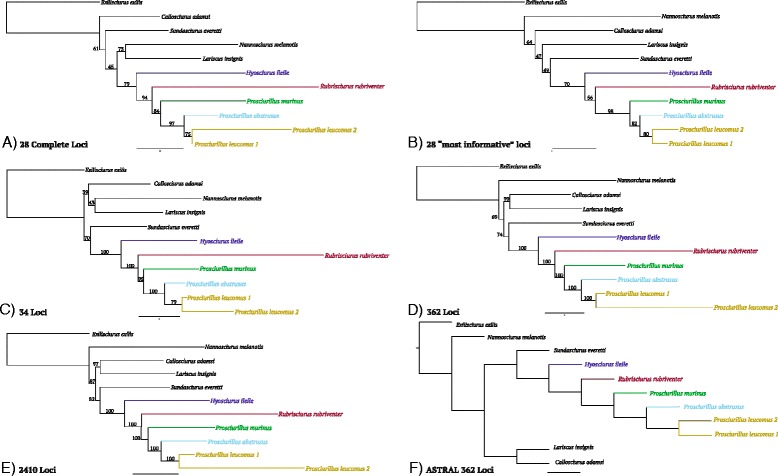
Comparative analysis of UCEs, with several trees provided based on various partitioning schemes. All trees were generated with Maximum Likelihood via PHyML except F. **a** the complete matrix of 28 loci, (**b**) a tree of the 28 ‘most informative’ loci, including loci with the highest number of informative sites per locus. **c** a tree of 34 loci where *Prosciurillus murinus* and *P. abstrusus* were always present, (**d**) the tree containing 362 loci, which included at least 9 of the 11 taxa, and at least three informative sites per locus. **e** A tree of 2410 loci containing at least 9 of 11 taxa and at least a single informative site per locus. **f** ASTRAL species tree estimation, generated from the 362 loci dataset. Additional details about partitioning are found in the Methods

*Prosciurillus murinus* (USNM 217817) had the fewest number of enriched UCE loci within *Prosciurillus* (1067), with data missing for a large percentage of loci in the incomplete dataset analyses. The placement of *Prosciurillus abstrusus* varied depending on the amount of information per included locus. To provide better resolution for the placement of these two species, and to reduce the effects of missing data on topology and branch lengths, another analysis was done including 34 loci (12,860 bps) in which *P. murinus* and *P. abstrusus* were always included. In order to evaluate these various data partitions, PHyML [38] runs were performed (all with HKY substitution models) on each of the above data partitions, as well as a species tree inference via ASTRAL [39] (Fig. 4) and NJst [40] (Additional file 2: Figure S1).

The topological relationships for ingroup taxa remained the same regardless of how the data were parsed. Length, number of informative sites, and number of differences per UCE locus were plotted to visualize the effects of reducing the dataset (Additional file 3: Figure S2). Relationships between outgroup taxa varied, but in five of the six trees (from Fig. 4a-f) the genus *Sundasciurus* (represented by *S. everetti* [41]) was recovered as the sister lineage to the radiation of Sulawesi squirrels. Similar to the mitochondrial results, within the Sulawesi radiation *Hyosciurus* was the earliest diverging lineage with *Rubrisciurus* and *Prosciurillus* recovered as sister genera. Within *Prosciurillus*, *P. murinus* was the most divergent lineage. Branch lengths were variable, especially for *Prosciurillus leucomus* 2 (USNM 200274), which was due to missing data.

**Fig. 5:**
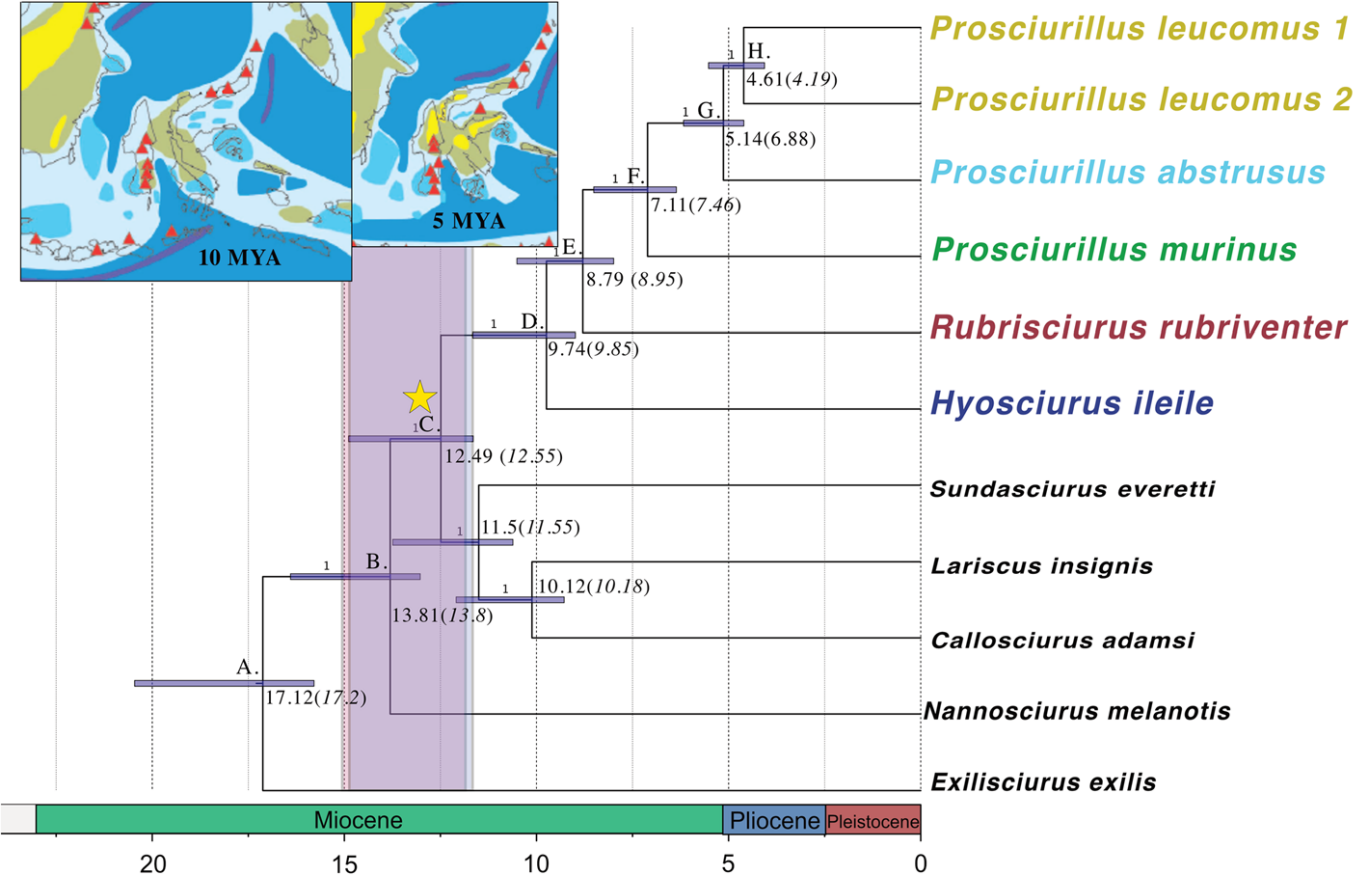
Divergence dated UCE dataset with two independent analyses (see methods) shaded (95 % CI) in different colors (although almost entirely overlapping). The inlaid maps are geological reconstructions from [8], reprinted with permission, highlighting the approximate conformation and extent of subaerial land at 10 and 5 MYA. The *green* indicates land, *yellow* indicates highland habitat, *red triangles* represent volcanoes, and the *shades of blue* represent shallow-deep sea (*light–dark blue*)

Both the concatenated (using PHyML [38] and MrBayes [42]) and the species tree inference methods (using ASTRAL [39] and NJst [40]), which consider the loci independently, yielded the same topology (ASTRAL, Fig. 4f; NJst, Additional file 2: Figure S1). The 362 loci dataset was dated with various partitioning schemes and substitution models. Each of the four replicates for the BEAST analysis converged and had robust ESS values (>200). Estimated divergence times were relatively consistent across datasets, with an estimated most recent common ancestor at 12.5 MYA for the 362 loci analysis (HKY substitution model), and 12.7 MYA (HKY + G), and 12.6 MYA for the partitioned analysis (Fig. 5, Table 3).

**Table 3:**
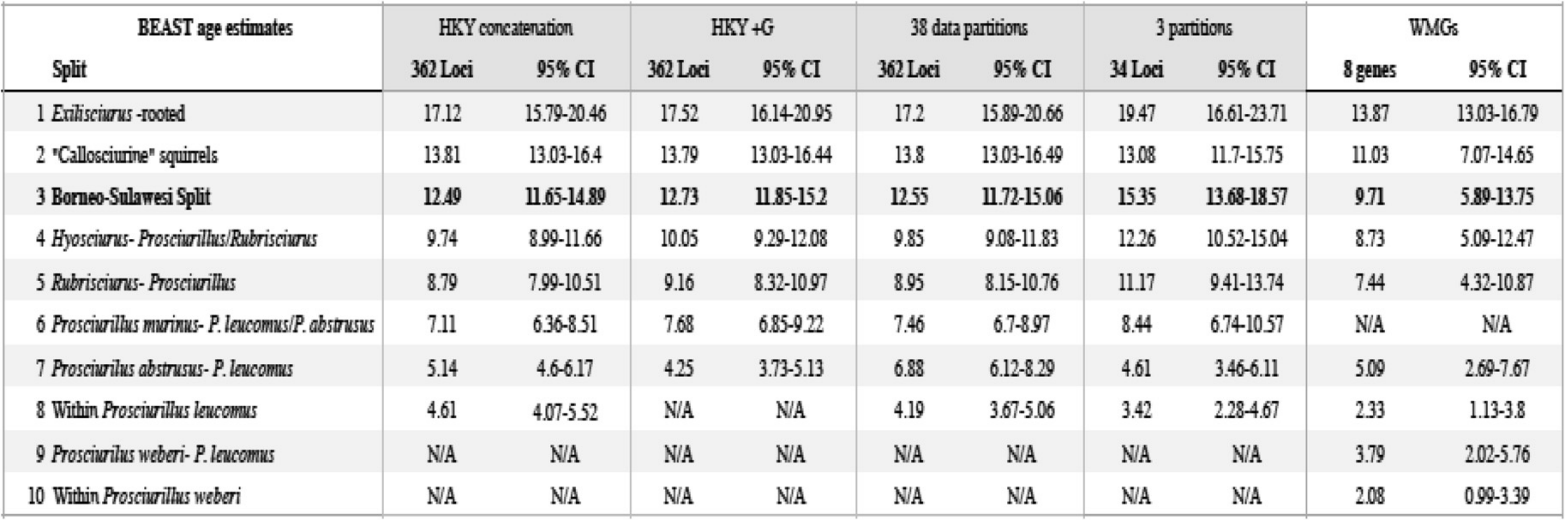
Comparison of BEAST divergence date estimates between both the mitogenome and UCE datasets. Several analyses of the UCE dataset are shown. Various dates from UCE partitions are shown, with the associated columns shaded in grey. The split between extant Bornean and Sulawesi taxa is in bold (Split 3)

## Discussion

### UCE characterization in mammals

As the number of reported cases of incongruence between gene trees and species trees increases, it is imperative to consider species level phylogenetic analyses based on evidence from multiple, independently inherited markers [43]. In early reports of incongruence it was unclear which of the small number of gene trees best reflected the species tree [43, 44]. However, now that a large number of independent nuclear loci can be analyzed, highly accurate species trees can be constructed and the vagaries of lineage sorting in individual genes ceases to be an issue [45]. UCEs are a large panel of nuclear markers developed across vertebrates that can be used to overcome the limitations imposed by small numbers of genes and have been shown to be useful in reconstructing species trees at various evolutionary scales [32, 34, 46, 47]. Here we used the same loci to demonstrate their power for resolving intergeneric relationships among Sulawesi squirrels. Our data suggest that these loci may be useful in a wide variety of animals at a similar evolutionary scale (i.e., diversification within and between mammalian genera) [33, 34, 48].

In this study, a large portion of our results was generated from degraded DNA extracted from museum specimens. Amplifying the entire mitochondrial genome and minimally 1000 nuclear loci in small overlapping fragments followed by Sanger sequencing would have been technically challenging and cost prohibitive. We have demonstrated the effectiveness of enrichment protocols, such as for the UCEs and mitogenomes, in degraded samples. Our lower quality museum samples enriched fewer loci than the high quality frozen tissue samples, but despite this, over 1000 UCE loci per sample (with an average length of 432 bp per locus) were recovered in all but one case. We observed high variability in the number of recovered UCE loci from the museum specimens, from 286 (*Hyosciurus heinrichi*) loci to 3800 (*Nannosciurus melanotis*), with an average of 810 loci per museum specimen (which is significantly lowered due to the low number of loci captured in *Hyosciurus heinrichi*; the average without this sample was 2939 loci).

Since most UCE loci represent DNA fragments of unknown function, here we implemented the *k*-means algorithm shown to produce better partitioning schemes than traditional methods (e.g. by locus, codon position, etc.) [49]. Robust phylogenies were generated from both coalescent and non-coalescent approaches, yet individual loci recovered a variety of topologies, dependent on the included taxa, number of informative sites per locus, amount of missing data and of course, lineage sorting.

### Evolutionary history of the Sulawesi squirrels

Sulawesi was never connected by land to Borneo, so an overwater ‘sweepstakes’ crossing is the most likely mechanism by which squirrels arrived on Sulawesi. Our estimated time of divergence between Sulawesi and Sundaland squirrels ranged from ~9.7–12.5 MYA on average including both the mitogenome and UCE datasets respectively, compared to divergence estimates of 10.5–11.4 MYA reported by Mercer and Roth [16]. All of our divergence time estimates from UCEs (using a strict molecular clock) resulted in 95 % HPD outside of, and older than the estimates from Mercer and Roth [16] (~12.5 MYA average, 11.65–15.2 MYA 95 % HPD from the UCE dataset), whereas our mitogenome dataset yielded a younger estimate of 9.71 MYA (95 % HPD: 5.89–13.76 MYA). The difference is likely due to the use of a relaxed molecular clock with the mitogenome dataset. Our results suggest that squirrels colonized Sulawesi during the Miocene, likely to west Sulawesi, which was located further south and west of its present location. The east and central peninsulas may have been partially connected at this time, but the other peninsulas were not [6, 50]. Based on the observed topology and monophyly, our results also support a single colonization event, as proposed by Mercer and Roth [16].

The deep divergence times between the three genera predate the modern configuration of Sulawesi by several million years. The three extant Sulawesi genera diverged from the outgroup taxa about ~9.7–12.5 MYA—a divergence that may potentially be linked to sea level changes during this timeframe [5, 16, 51]. Low sea level may have facilitated a scenario of initial rafting to Sulawesi, followed by allopatric diversification with squirrel lineages diversifying on different islands/areas that subsequently came together to form Sulawesi.

A few million years after the original crossing to Sulawesi, mountain uplift created the first highlands in the region [8], and potentially allowed for additional niche specialization in Sulawesi squirrel species. Hall [8] suggests the inclusion of biological and molecular evidence to deduce the precise timing of evolution in Sulawesi, as the geologic record is incomplete. The endemic squirrels represent a group of completely terrestrial, forest-dependent mammals, which are known as poor over-water dispersers. We propose that the divergence dates observed in endemic squirrels are likely closely tied to the geologic events − as originally described by Merecer and Roth [16] − because several species are restricted to specialized habitats (including montane, mangrove, and peninsula restricted species).

Specifically, our divergence estimates based on mtDNA for *Hyosciurus heinrichi* and *H. ileile* are dated at approximately 5.76 MYA, coinciding approximately with the timing of uplift in the central core of Sulawesi. Within *Prosciurillus*, *P. abstrusus* (a montane endemic) was dated as splitting from other *Prosciurillus* species between 5.09 and 5.14 MYA (mitogenomes and UCEs respectively), which is also likely tied to the uplifting and/or isolation of this species in the mountains of the south peninsula [8]. *Prosciurillus leucomus* was estimated to have diverged between 2.3 and 4.6 MYA (mitogenomes and UCEs respectively), which is likely near the time when the northern peninsula connected to the central core of Sulawesi [8]. Finally, *Prosciurillus weberi*, a species currently restricted to mangrove forests in the southernmost extent of the central core of Sulawesi, was estimated to have diverged 3.79 MYA, with over two million years of history within the species, and may provide an indication of the formation or expansion of mangroves in Sulawesi around this time.

We note that our dating estimates should be taken with caution because they were recovered from fossil calibration points far outside of the Sulawesi taxa, and we did not use geological data to constrain divergence dates. By combining the known geological record with carefully dated phylogenetic trees, we can begin to reveal finer scale patterns of speciation and movement across this biodiversity hotspot. The inclusion of additional species and large datasets can provide an independent source of information regarding the estimated convergence of all land fragments in Sulawesi, and provide further evidence for a better resolution of the evolutionary history of this endemic radiation within Sulawesi.

### Discordance between mitochondrial and nuclear datasets

The well-supported monophyletic groups resulting from the species tree analysis are consistent with previous generic level designations based on morphology. Discordance between mitochondrial and nuclear datasets has been well-documented in animals [52]. Since mitochondrial DNA is inherited as a single unit, it reflects only the genealogical history of a single locus and hence phylogenies derived from mtDNA result in gene trees, which may or may not be congruent with the species tree. However, examination of this marker (when compared with multiple unlinked loci) can provide insight into evolutionary processes (e.g. introgression, selection, drift) that have influenced phylogenetic patterns.

Considering the estimated age of this group, the paraphyletic relationships in *Prosciurillus* observed in the mitochondrial dataset are likely the result of an ancient mitochondrial introgression event. Mitochondrial introgression has been reported in a variety of animals and occurs more frequently in mtDNA compared to nDNA (see Ballard and Whitlock [53] and citations within). Mitochondrial introgression into the *Prosciurillus murinus* lineage is best explained by ancient introgression when a population of ancestral *P. murinus* expanded its range into the range of another lineage not sampled here. Some of the oldest events of ancient introgression of mtDNA have been reported in squirrels [54–56]. Simulations suggest that introgression almost exclusively occurs in the direction from the native to the invading species [52], as also empirically illustrated by some late Quaternary expansions [57, 58]. That implies that when a proto- *Prosciurillus murinus* lineage colonized a new island/region up to 7 MYA (which corresponds to the most recent age estimate based on UCE’s of *P. murinus* from other Sulawesi species, after splitting from other species), it encountered and reproduced with squirrels already present there. The other lineage of squirrels was not sampled in this study, possibly because it went extinct after the arrival of *Prosciurillus*, but their mitochondrial lineage became fixed in the expanding species. The review of mito-nuclear discordance presented by Toews and Brelsford [44] found that 97 % of surveyed studies detailed situations where isolation had occurred, followed by secondary contact leading to the introgression events, which may be the most likely scenario for the patterns observed in *Prosciurillus*.

Relatively short internodes in the deeper parts of the phylogeny can be problematic for species tree construction (i.e. between *Hyosciurus*, *Prosciurillus* and *Rubrisciurus*). Studies of adaptive radiations that occurred over short evolutionary time scales (e.g. African cichlid fishes) have reported difficulty in resolving nodes deep at the base of the radiation [59]. As demonstrated here and elsewhere, large genomic datasets provide enough power to resolve these difficult nodes. However; these results need to be taken with caution as large datasets can also potentially inflate support values when gene sequences are concatenated, leading to higher confidence in incorrect phylogenetic trees [45, 60–63]. The initial rapid diversification rates (combined with short branches between lineages) of adaptive radiations likely make the deep nodes very difficult to resolve, and by increasing data with HTS, branch support could be misleading [63, 64]. In this system, the different topologies observed between coalescent and non-coalescent analyses of our mitogenome dataset (placement of *Rubrisciurus* and *Hyosciurus* with respect to *P. murinus*) may be linked to the short internodes between genera with fewer informative sites to properly place these lineages or violations of model assumptions. Our results provide further justification for using many unlinked loci for resolving phylogenies. In this study, the results based on mitochondrial data alone would have questioned the monophyly of the genus *Prosciurillus*.

While contamination is a major concern when working with degraded DNA from historical specimens [65–67], the observed discordance between mitochondrial and nuclear datasets in this study is clearly due to evolutionary processes rather than to problems with contamination. If the result were due to contamination, we would expect to see issues with both the mitochondrial and nuclear datasets because DNA samples for both marker analyses were conducted on the same extraction aliquots. Second, we replicated library preparation and in-solution hybridization protocols, which resulted in confirmation of the results presented here. We also followed steps to identify and ensure removal of nuclear copies of mitochondrial DNA in our samples [35, 68]. Finally, cross-contamination would have resulted in heterozygosities in the mitochondrial dataset, and either admixed placement of the samples in the UCE analyses, or poor support for these nodes. The only cases where we detected evidence of contamination involved samples from two museum specimens from tubes that were damaged during transport. These samples were excluded from all analyses.

### Conservation implications

The data presented here unveil deep divergences within species, from 1.28 million years within *Rubrisciurus rubriventer*, to 2.33 million years between subspecies of *Prosciurillus leucomus*. Even the mangrove-restricted species *Prosciurillus weberi* showed over 2 million years of divergence between individuals, indicating significant genetic diversity in a limited geographic range. These results highlight the evolutionary distinctiveness of the isolated populations of squirrels found in this highly threatened landscape. More exhaustive sampling across the range of individual species could reveal additional ancient lineages, and perhaps allow us to determine the level of divergence within and between all species.

Evans et al. [69] suggested conservation priorities for seven areas across Sulawesi for which macaques and toads had similar species distributions. The squirrels are not tightly correlated to the same seven regions, and seem more habitat specific (with mangrove and montane specialists), which may, for example, instead explain the similarity to patterns revealed in species of viviparous freshwater snails [50]. An ecosystem approach might be a better method of determining conservation priorities to encompass the differences observed in faunal distribution across Sulawesi, but more research, ideally involving well-sampled phylogenies across a much wider diversity of Sulawesi biodiversity, is needed to address this question and ultimately to provide the best-informed conservation plan and practices.

## Conclusion

The additional taxonomic sampling of Sulawesi squirrels and outgroup taxa from Sundaland obtained from museum specimens coupled with sequence capture of a large number of unlinked UCE loci and mitogenomes resulted in a well-resolved phylogeny that dates the divergence of endemic Sulawesi squirrels from their closest relatives to ~9.7–12.5 MYA. Our results confirm the monophyly of Sulawesi squirrels with deep divergences between the three endemic genera and support a single colonization event that likely occurred during the Miocene and represents an old adaptive radiation that predates the amalgamation of the current island of Sulawesi. The incorporation of novel HTS technologies and newer geological models are leading to new biogeographic insights and further understanding of the evolutionary processes driving adaptive radiations in this region. These biogeographic insights and the patterns of faunal exchange in and out of Sulawesi make this island an extremely interesting place for future research. Also, we found incongruent evolutionary relationships derived from mitochondrial and nuclear markers which we hypothesize are due to an ancient introgression event. Future research that incorporates dense taxon sampling and all nominal species is needed to completely understand the evolutionary history and allow us to test more fine-scale hypotheses concerning dispersal and diversification within this unique group of squirrels. Finally, as deforestation has reached unprecedented rates in this region, it will be important to implement an ecosystem approach to better determine conservation priorities that encompass the differences observed in faunal distribution across Sulawesi. Future research with well-sampled and well-resolved phylogenies from a large set of representative taxa from Sulawesi is needed to address this question and ultimately to provide the best-informed conservation plan and practices.

## Methods

### Samples

A total of 17 ingroup samples from the three target genera and 5 outgroup taxa were included in this study. All ingroup samples were derived from degraded museum specimens, obtained from bone fragments, skin clips, or adherent osteological material from specimen skulls (Table 1). Sampling of specimens followed strict protocols including changing gloves between each sample, as well as bleaching all work surfaces and utensils prior to each use. Three species of *Prosciurillus* were not included in this study (*Prosciurillus alstoni, P. topapuensis,* and *P. rosenbergii)* because it was not possible to obtain samples. All outgroup taxa were high quality tissue samples (except *Nannosciurus melanotis*, which was also from a degraded museum specimen, and was included to represent an additional extant Bornean genus).

Specimens were collected following ethical guidelines, detailed in [41], and approved from institutional animal care and use committees (Smithsonian Institution, National Museum of Natural History, Proposal Number 2012–04, and Estación Biológica de Doñana Proposal Number CGL2010-21524). Research permits from Sabah Parks were approved under permit number TS/PTD/5/4 Jld. 47 (25), and exported with permissions from the Sabah Biodiversity Council (Ref: TK/PP:8/8Jld.2). DNA extractions of museum specimens were performed by phenol/chloroform isolation in a specifically dedicated laboratory (for low quality ancient DNA) that is physically separated from the main laboratory. To monitor for contamination, negative extractions were included in every batch of extractions, library preparation was replicated, and sequencing was performed in multiple laboratories. Samples were subsequently concentrated via centrifugation [70]. Tissue samples for the outgroups were extracted with Qiagen DNeasy Blood and Tissue Kits (Valencia, CA) and eluted in 200 μl of Qiagen elution buffer. All samples were subsequently stored at −20 °C.

### Library preparation

Several library preparation methods were used, including a modification of Roche 454 library preparation for Illumina sequencing, which is detailed in the supplemental materials. Commercial kits were used for both museum and tissue samples, as detailed in [71]. After successful library preparation, amplification of indexed libraries was performed with a high fidelity *Taq* (either Phusion HF *Taq* or Kapa HF *Taq*) for 18 cycles on museum specimens, and 14 cycles on modern tissue samples.

### Mitochondrial genome generation

#### Museum samples

Due to the degraded nature of the samples and variation in copy number of mtDNA versus nDNA, separate enrichments were performed for each type of DNA and museum versus frozen tissue samples. For mitogenomes generated from museum specimens, two to four samples were multiplexed and enriched following the protocol described in [71, 72].

#### Tissue samples

As the frozen tissue samples were high molecular weight samples, enrichment of mitogenomes was not required, and instead long-range PCR (LR PCR) amplified the entire mitogenome in two fragments with universal primers [73, 74]. Takara LA *Taq* (Clonetech) was used for LR PCR, and amplified for 35 cycles with a 68 °C anneal. PCR products were then sonicated to randomly shear the PCR products. A QSonica Q800RS was used for sonication using 25 % amplitude with an on/off pulse for 5 min. Products were subsequently visualized on an agarose gel to evaluate the resulting fragment size. After magnetic bead purification (MagNA) following Rohland and Reich [75], Kapa library preparation kits were used for library preparation of approximately 500 ng of template DNA.

### Ultraconserved elements

Museum and tissue samples were enriched for UCEs in multiplexed pools of eight samples (combined with samples from other unrelated projects). Reduced subsets of individuals were enriched for UCEs due to the cost to enrich and sequence these samples. No museum samples were multiplexed with modern tissue samples to prevent biased enrichment of the samples. Reduced sets of specimens were enriched for UCEs due to the anticipated allelic dropout with museum samples and deeper sequencing requirements. The UCE enrichments were performed with DNA based probes using the NimbleGen SeqCap EZ (Roche) kit. The probe set contained approximately 4000 UCE loci designed for tetrapods [46]. After enrichment, amplification was performed (following the manufacturer’s protocol). Enrichments were visualized after MagNA purification on both an agarose gel and on the Bioanalyzer (Agilent, High Sensitivity Kit). Problems with removal of adapter dimer were common with the museum samples, as the target DNA and the adapter dimer were fairly close in size; so complete removal of adapter dimer was rarely possible. To compensate for this, enrichments with dimer were sequenced at higher coverage.

### Quantification and sequencing

Following visualization of the enrichments, quantitative PCR (qPCR) was performed using SYBR green florescence (Kapa Biosystems Illumina Library Quantification Kit). Equimolar pooling of samples was based on the values generated from qPCR. Illumina sequencing was done on either the Illumina MiSeq or HiSeq 2000 with either 2 × 250 bp or 2 × 150 bp paired end sequencing, respectively. Since the coverage requirements were substantially different for the mitogenomes versus the UCEs, the two types of enrichments were sequenced on separate runs. Sequencing was performed at the Semel Institute of Neurosciences (UCLA), National High-throughput DNA Sequencing Centre (University of Copenhagen, Denmark), and the Center for Conservation and Evolutionary Genetics (Smithsonian Institution, National Zoological Park).

### Data analysis

Raw fastq files were generated from sequencing cores for both mitogenomes and UCEs. The processing of the two molecule types (mtDNA versus nDNA) was done separately. For the mitogenome analysis, an average of 1,041,190 merged reads were recovered for each sample, with individuals ranging from 25,633 to 9,706,968 per individual (see Table 2). The samples screened for UCEs recovered an average of 3,571,092 paired end reads, ranging from 771,645 to 9,891,988 (see Additional file 4: Table S2).

### Mitogenome analysis

Both museum and tissue samples were analyzed with the same pipeline, with the modern samples skipping read merging. Paired-end reads for each museum sample were merged together using the program PEAR v.0.9.4 [76] to allow for better downstream read mapping. Residual adapter fragments were removed with the program Cutadapt v1.4.2 [77]. Reads with a mean quality score below 20 and exact PCR duplicates (three or more identical molecules) from the 5′ direction were removed with Prinseq v.0.20.4 [78]. The high quality reads were then mapped to a reference sequence. There are no previously published mitogenome sequences for any of the endemic Sulawesi squirrels, so a consensus of three Sundaland squirrels was used as a reference: *Callosciurus erythraeus* (Accession # NC_025550.1), *Sundasciurus everetti* (Accession # KR911798; [41]), and *Lariscus insignis* (generated here, Accession # KR911799). This reference is referred to in Table 2 as 'Sulawesi Reference'. The cleaned reads were mapped to the consensus sequence with bwa v.0.7.10, using the ‘bwa mem’ command [79]. The resulting SAM file was imported to Geneious v.7.1.7, visually inspected, aligned to other mitogenomes, and subsequently annotated.

### UCE analysis

A total of 12 samples were included in the UCE analysis. These contained eight endemic Sulawesi taxa and five outgroup taxa. The complete bioinformatic analysis of the UCE dataset followed the published pipeline phyluce [48], available at http://phyluce.readthedocs.org/en/latest/. The phyluce pipeline has options for generation and analysis of both complete and incomplete UCE matrices, and both were generated here. In order to determine which loci informed phylogenetic relationships, a script from the phyluce pipeline was run to determine the number of informative loci from both the complete and incomplete dataset. A number of comparisons were made between the complete, and subsets of the incomplete, matrix. In addition to the standard phyluce pipeline, MARE [80] was used to test for biases in matrix reduction, and FASconCat-G [81] was used to concatenate the loci for phylogenetic analysis, where appropriate.

### Phylogenetic analysis

#### Mitogenomes

MEGA v.5.2.2 was used to test for the molecular clock on both the ingroup, and the entire dataset including outgroups, for which a strict clock was rejected in both instances [82]. In order to determine the amount of saturation, all protein coding genes were extracted from the mitogenomes, and transversions were plotted against transitions between codon positions 1,2 versus 3. PartitionFinder was used to determine the codon partitioning and substitution model for each mitochondrial gene [83]. Phylogenetic trees were generated with both maximum likelihood (ML) and Bayesian inference (BI) approaches. PhyML [38] was run with the Geneious v.7.1.7 plugin for the ML tree; 100 bootstrap replicates were run with the non-partitioned concatenated dataset. The substitution model used was HKY and the topology was optimized for tree topology, branch lengths, and substitution rates. MrBayes v3.2.3 [42] was used to generate the BI tree, and three independent runs were completed for 2 million chains, with a subsample frequency of 1000, and four heated chains at a temperature of 0.2. Independent runs were evaluated for convergence. Unconstrained branch lengths were allowed, and the first 100,000 trees were discarded as burnin. The average standard deviation of split frequencies (ASDSF) was evaluated to determine the convergence between runs. The non-saturated mitochondrial genes (CO1-3, ND1-5) were included in a divergence dating analysis using BEAST v.1.8.1. [37] PartitionFinder [83] was used to determine the codon partitioning and substitution model for each non-saturated gene. A lognormal (uncorrelated) relaxed clock was used under a Yule speciation tree, with operator left to default. A single fossil calibration was used for the *Callosciurus* + *Sundasciurus* + *Nannosciurus* + *Lariscus + Exilisciurus* clade of squirrels, which here included only the outgroup sequences (*Sundasciurus everetti*, *Callosciurus adamsi, Nannosciurus melanotis, Lariscus insignis, and Exilisciurus exilis*) based on first observance of *Callosciurus* as well as fossils of *Dremomys* and *Tamiops* [84]. The specific range for the *Callosciurus* fossil (focused on here as there is evidence for earlier *Dremomys* and *Tamiops,* which are not included in this dataset) is from 14.3–13.2 MYA, which was recovered from the lower part of the Chinji formation (Lawrence Flynn, pers. comm.). Older fossils representing *Dremomys* and *Tamiops* are available at this same site, and an ancestor to *Tamiops* has been recovered from around 20 MYA [84]. Due to the paucity of the fossil record, obtaining the best fossils for divergence dating can be difficult, and sometimes requires incorporation of fossils and inferred dates in combination, as done in [41]. A lognormal distribution was centered at 14.0 MYA and the 5 and 95 % quantiles were 13.19 and 18.18 MYA respectively. We allowed for additional depth past the 14.3 MYA to account for some degree of discrepancies in the fossil record, and to coincide with the topology and date ranges found in the two previous sciurid phylogenies, particularly to incorporate the placement of *Exilisciurus* [16, 85]. Four independent runs were performed to assess run convergence using Tracer v.1.5 and TreeAnnotator was used to combine individual runs [86].

#### UCEs

In order to determine the number of effective partitions, we ran the program PartitionFinder [83] on the Smithsonian Institution High Performance Cluster (SI/HPC). MEGA v.5.2.2 was used to test for the molecular clock, for which a strict clock could not be rejected [82]. Both Maximum Likelihood (ML) and Bayesian Inference (BI) trees were produced. PhyML [38] was run with the Geneious v.7.1.7 plugin, and MrBayes v. 3.2.3 [42] was run on XSEDE via the Cipres Science Gateway [87]. The BI analysis was run three times with 10,000,000 generations along four chains with two replicates at a temperature of 0.05. The ASDSF was evaluated to determine the convergence between runs. Sample frequency was set to 100 with a burnin of 10,000. Tree topologies were visualized with FigTree v.1.4 [88].

### Species tree inference

In order to evaluate the effects of different species tree methods, we ran the 362 UCE loci dataset with ASTRAL4.7.7 [39] and NJst [40] on the STRAW webserver [89]. ASTRAL employs a statistical binning approach to generate consensus trees for multiple genes, then combining them for a species tree [39]. NJst uses a distance method for inferring species trees from unrooted gene trees by neighbor-joining methods built from a distance matrix [40].

### Divergence dating

BEAST v.1.8.1 [37] was used to estimate the age of the endemic Sulawesi squirrels. The 362 UCE loci dataset was concatenated and run using a strict molecular clock with BEAST. One replicate was not partitioned, and included a substitution model of HKY. A second analysis incorporated HKY + G for the single partition. The *k*-means partitioning algorithm was used for the dataset containing the 362 UCE loci [49] and implemented through PartitionFinder [83]. This algorithm considers nucleotides separately, and not from locus information. Partitions as found from PartitionFinder were extracted with RAxML (using the –f s and –q options) [90]. The Yule process of speciation was used, and all operators were left to default. The same fossil calibration prior was used as described above. Four independent runs of BEAST were performed for each partitioning scheme and substitution model to assess for convergence between runs, which was done with Tracer v.1.5 [86]. Finally, an empty alignment was run to evaluate the effect of the priors on the data, which was also evaluated with Tracer v.1.5 [86].

### Ethics

All research involving animals followed institutional approval, and conformed to ethical guidelines (Smithsonian Institution, National Museum of Natural History, Proposal Number 2012–04, and Estación Biológica de Doñana Proposal Number CGL2010-21524). Research permits from Sabah Parks was approved under permit number TS/PTD/5/4 Jld. 47 (25), and exported with permissions from the Sabah Biodiversity Council (Ref: TK/PP:8/8Jld.2).

### Consent to publish

Not applicable.

### Availability of supporting data

All complete mitogenome sequences have been submitted to Genbank via the following accession numbers: KR911784-KR911801, KT001463.

The raw reads used to assemble UCEs have been uploaded to NCBI’s SRA under the Bioproject: PRJNA284011 and Biosamples: SAMN03658740, SAMN03658303, SAMN03658302, SAMN03658301, SAMN03658299, SAMN03658298, SAMN03658472, SAMN03658475, SAMN03658474, SAMN03658471, SAMN03658451. The raw reads were subjected to the phyluce pipeline available at: www.ultraconserved.org. All tree files have been placed on Dryad at http://dx.doi.org/10.5061/dryad.7v5p3.

## Additional files

Additional file 1: Table S1. Overall mean genetic distance across the 28 UCE loci in the complete dataset, as well as the 28 ‘most informative’ loci. The complete dataset is the first two columns, with the loci name, and overall mean distance (OMD) in the second column. The third and fourth columns are the ‘28 most informative’ loci. All calculations of overall mean distance were performed in MEGA v6.0 under default settings. (PDF 46 kb) Additional file 2: Figure S1. A species tree generated from NJst, which recovered the same topology as ASTRAL as well as the concatenated phylogenetic trees. (PDF 605 kb) Additional file 3: Figure S2. Graphs showing information from two subsets of the incomplete matrix of UCE loci. The left column contains all loci enriched (4046 total) and the right column shows all loci which contain at least three informative sites (1137 loci). The top graph shows the overall length distribution across all loci for both subsets, the middle shows the number of informative sites, and the bottom shows the total number of differences across the loci. Note the two show very similar patterns, which was justification to use a smaller, yet equally informative subset of all loci. (PDF 194 kb) Additional file 4: Table S2. Number of reads for each individual enriched for UCEs, and total number of recovered loci. (XLSX 48 kb)

## Abbreviations

HTS: high throughput sequencing
Mitogenome: mitochondrial genome
UCEs: ultraconserved elements
MYA: millions of years ago
ML: maximum likelihood
BI: Bayesian inference
