# Supplementary material for: Evolutionary history of endemic Sulawesi squirrels constructed from UCEs and mitogenomes sequenced from museum specimens
Source: BMC Evol Biol. 2016 Apr 14;16:80. doi: 10.1186/s12862-016-0650-z (PMC4831120; doi:10.1186/s12862-016-0650-z)
Supplement: Additional file 1: Table S1. — Overall mean genetic distance across the 28 UCE loci in the complete dataset, as well as the 28 ‘most informative’ loci. The complete dataset is the first two columns, with the loci name, and overall mean distance (OMD) in the second column. The third and fourth columns are the ‘28 most informative’ loci. All calculations of overall mean distance were performed in MEGA v6.0 under default settings. (PDF 46 kb) [file 12862_2016_650_MOESM1_ESM.pdf]

Table 1

	Species	Catalog #	# Loci	Raw # Reads UCEs
1	*Hyosciurus ileile*	ANMH 225461	2608	1946551
2	*Prosciurillus abstrusus*	AMNH 101360	3748	9891988
3	*Prosciurillus leucomus occidentalis 1*	AMNH 196571	3248	6517364
4	*Prosciurillus leucomus leucomus 2*	USNM 200274	1262	1657113
5	*Prosciurillus murinus 1*	USNM 217817	1067	7329386
6	*Rubrisciurus rubriventer 1*	USNM 218710	1359	771645
7	*Exilisciurus exilis*	ROM 102254	3706	2444601
8	*Callosciurus adamsi*	NZP95-322	3834	1528736
9	*Nannosciurus melanotis*	USNM 123098	3800	3964463
10	*Lariscus insignis*	MVZ192194	3838	1877547
11	*Sundasciurus everetti*	MTRB 3/ 16	3863	1352620
