# Supplementary figures and images for: Evolutionary history of endemic Sulawesi squirrels constructed from UCEs and mitogenomes sequenced from museum specimens
Source: BMC Evol Biol. 2016 Apr 14;16:80. doi: 10.1186/s12862-016-0650-z (PMC4831120; doi:10.1186/s12862-016-0650-z)

4046 UCE Loci

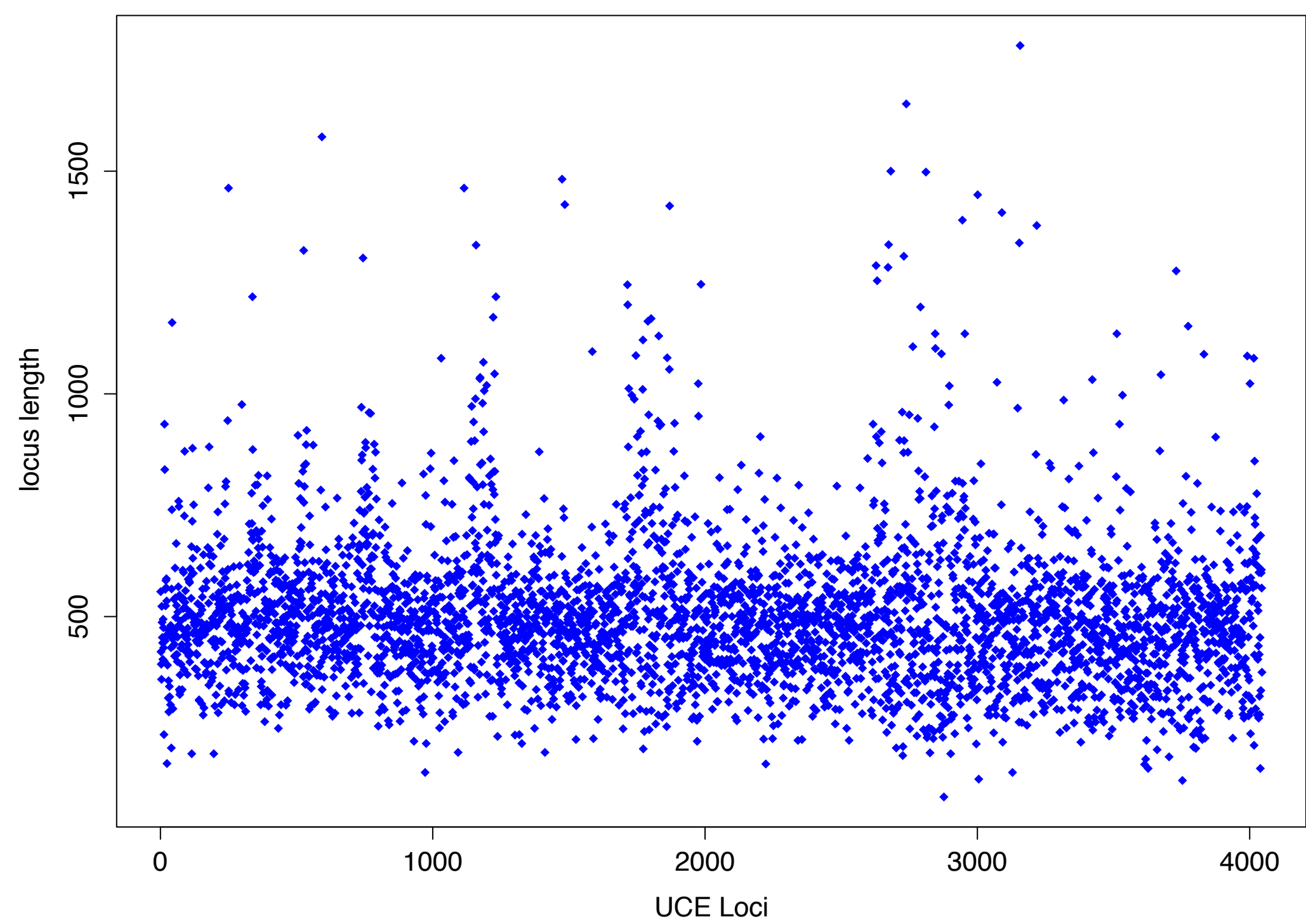

1137 UCE Loci

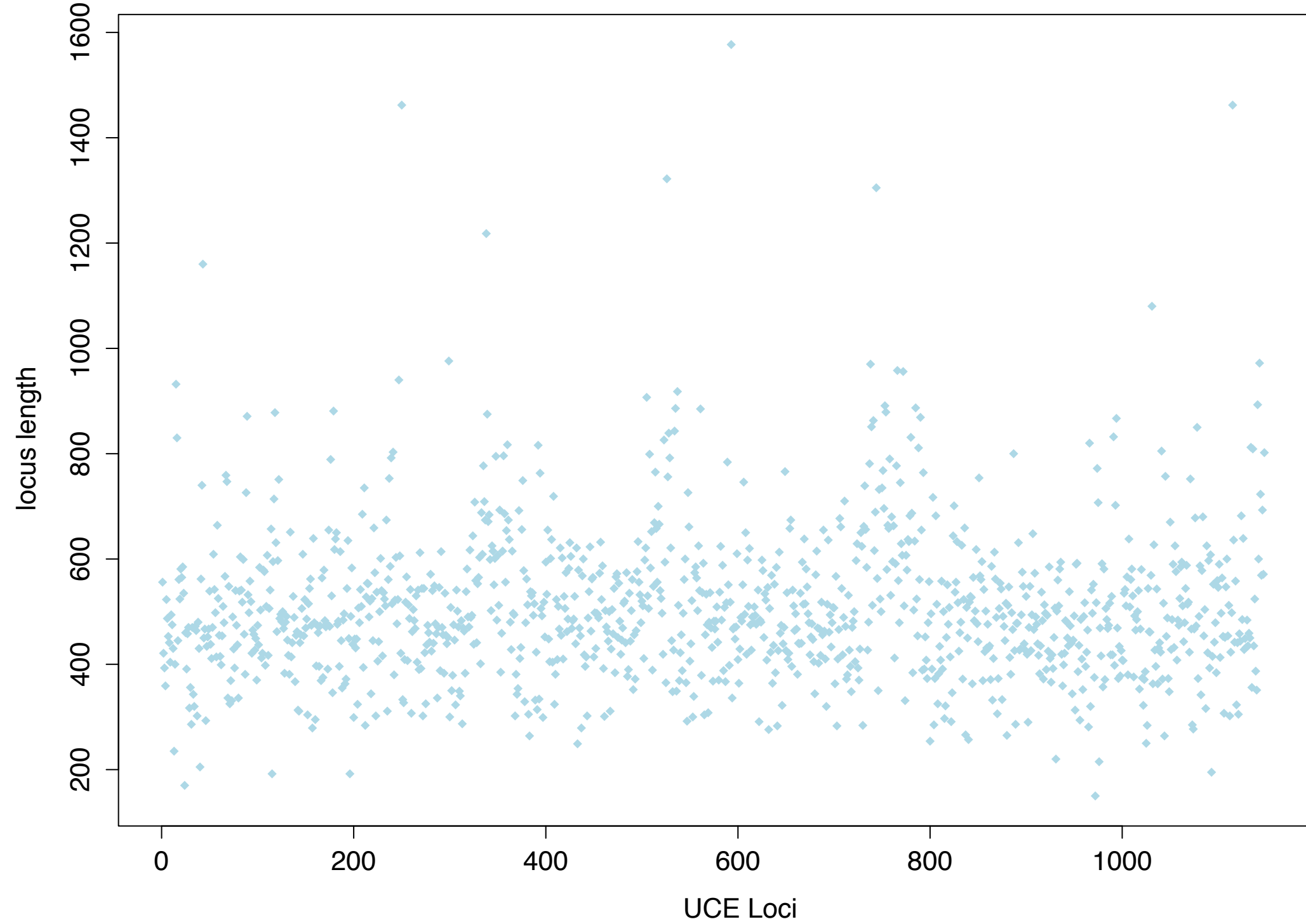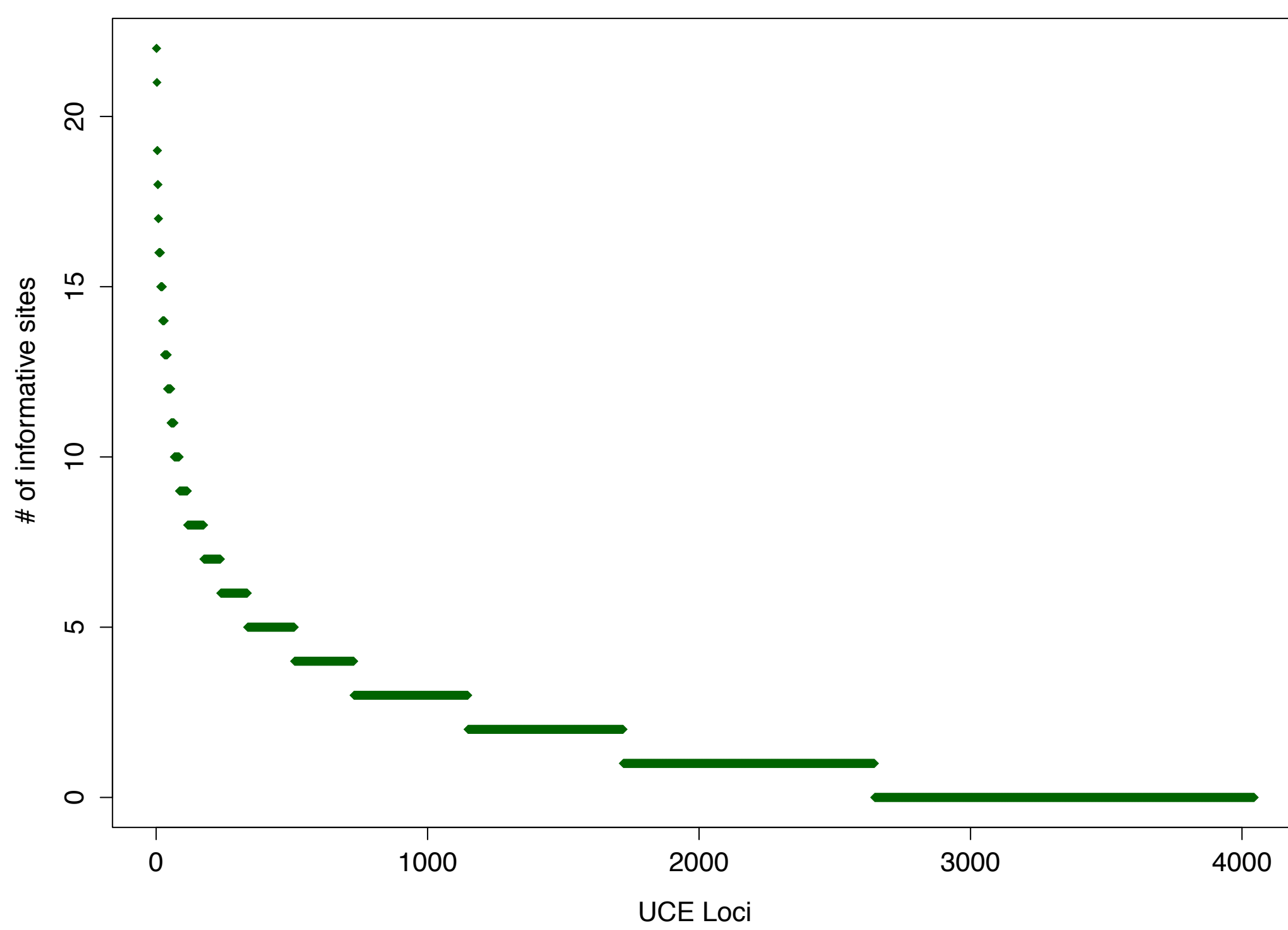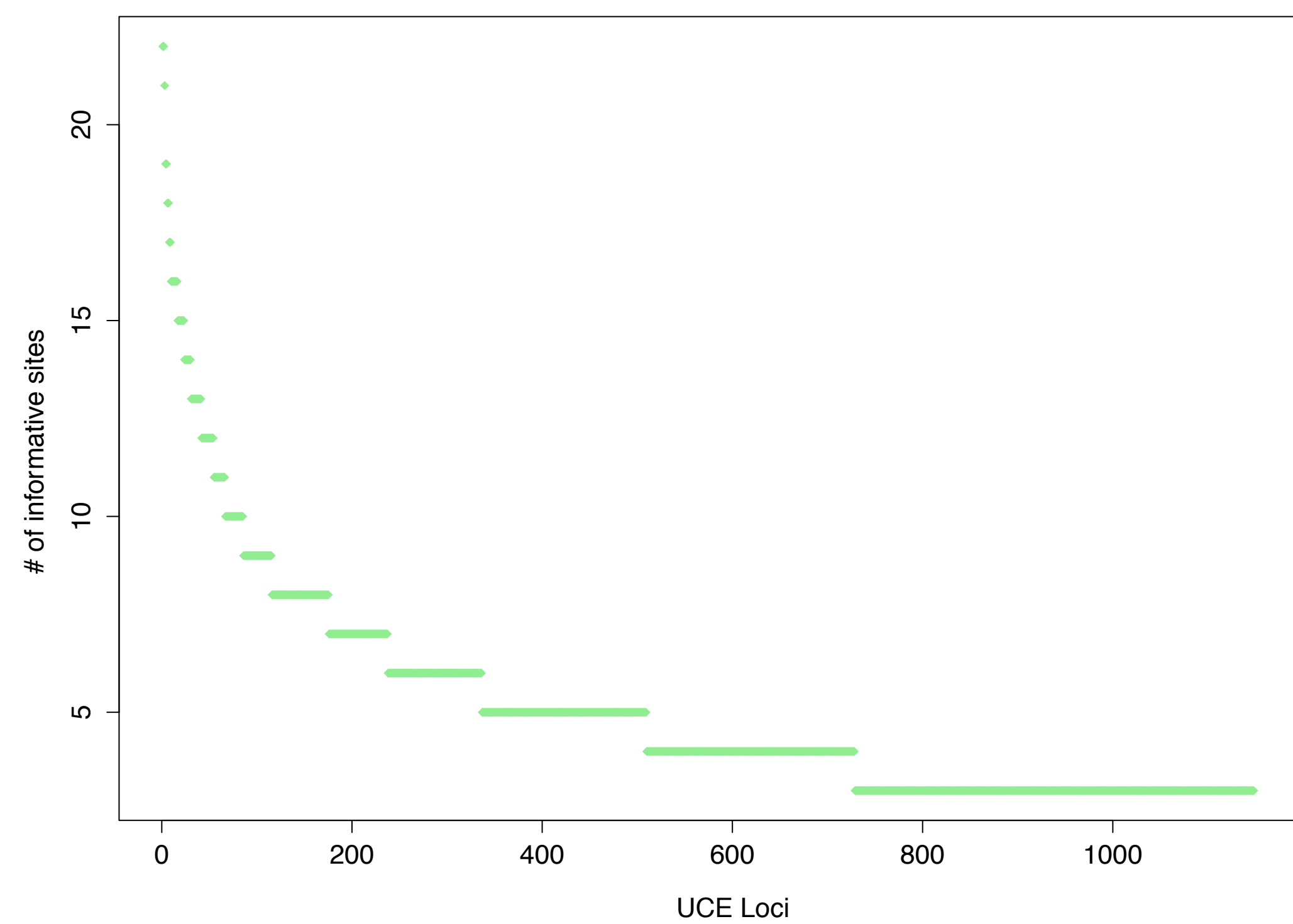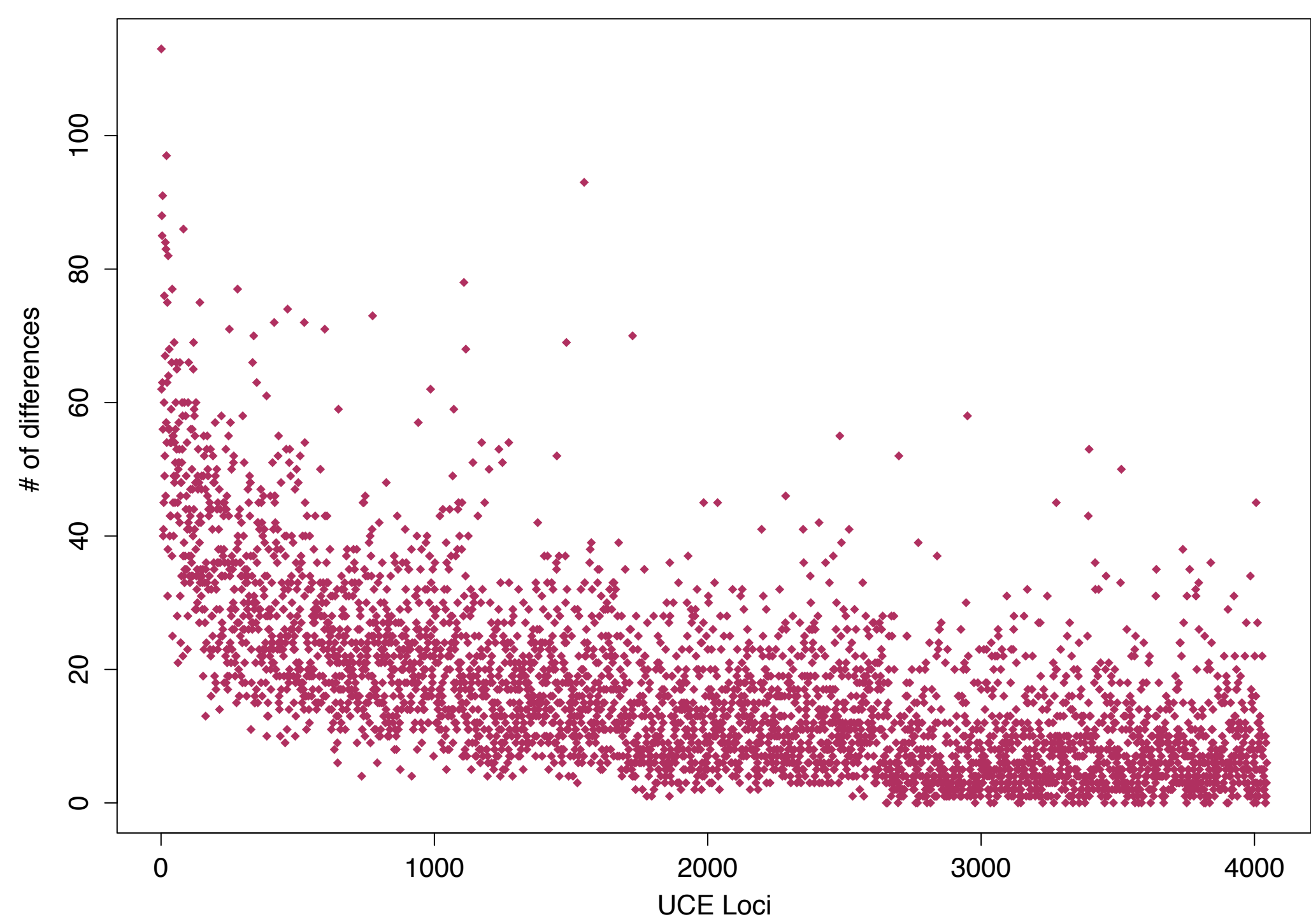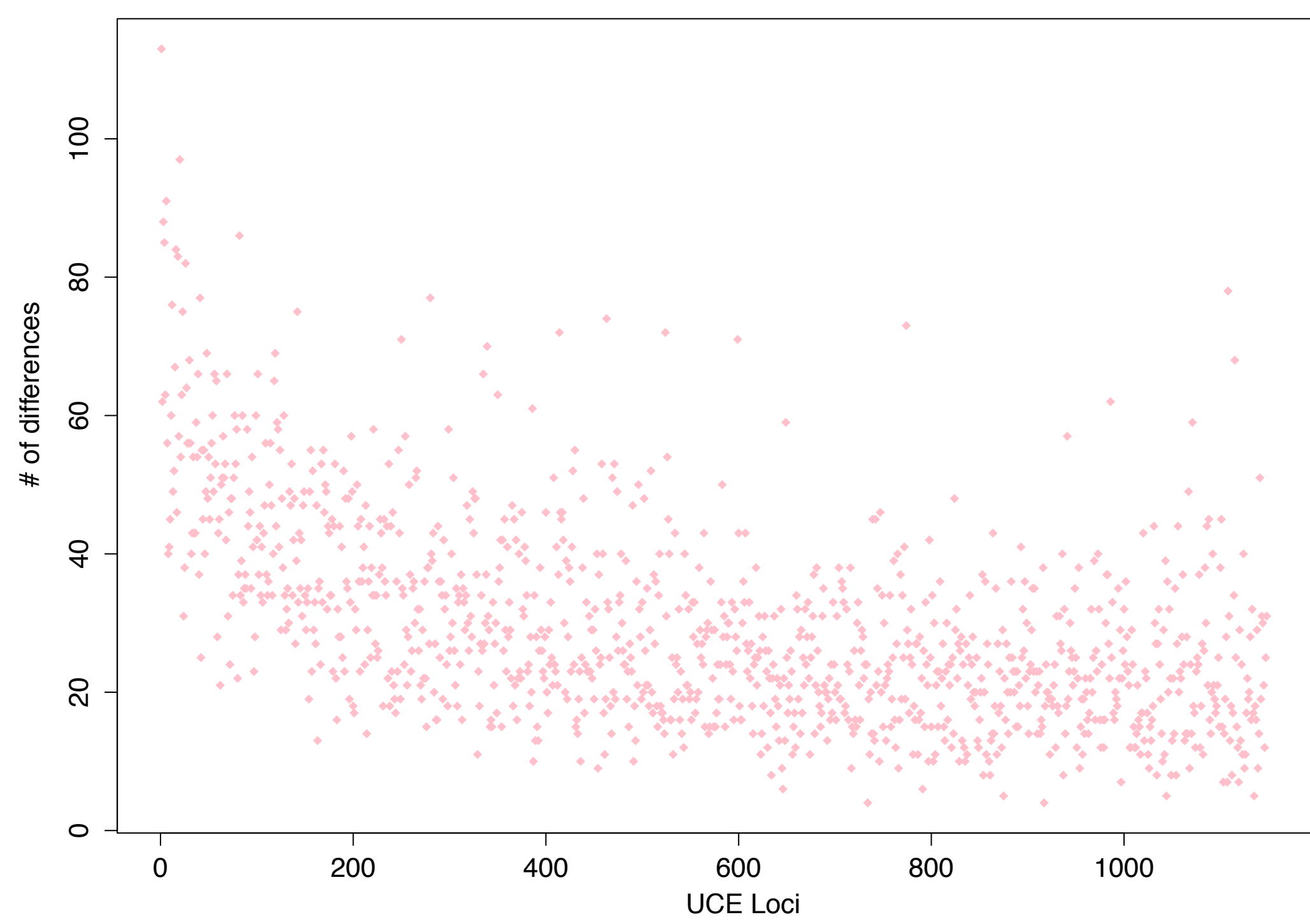

Supplement: Additional file 2: Figure S1. — A species tree generated from NJst, which recovered the same topology as ASTRAL as well as the concatenated phylogenetic trees. (PDF 605 kb) [file 12862_2016_650_MOESM2_ESM.pdf]

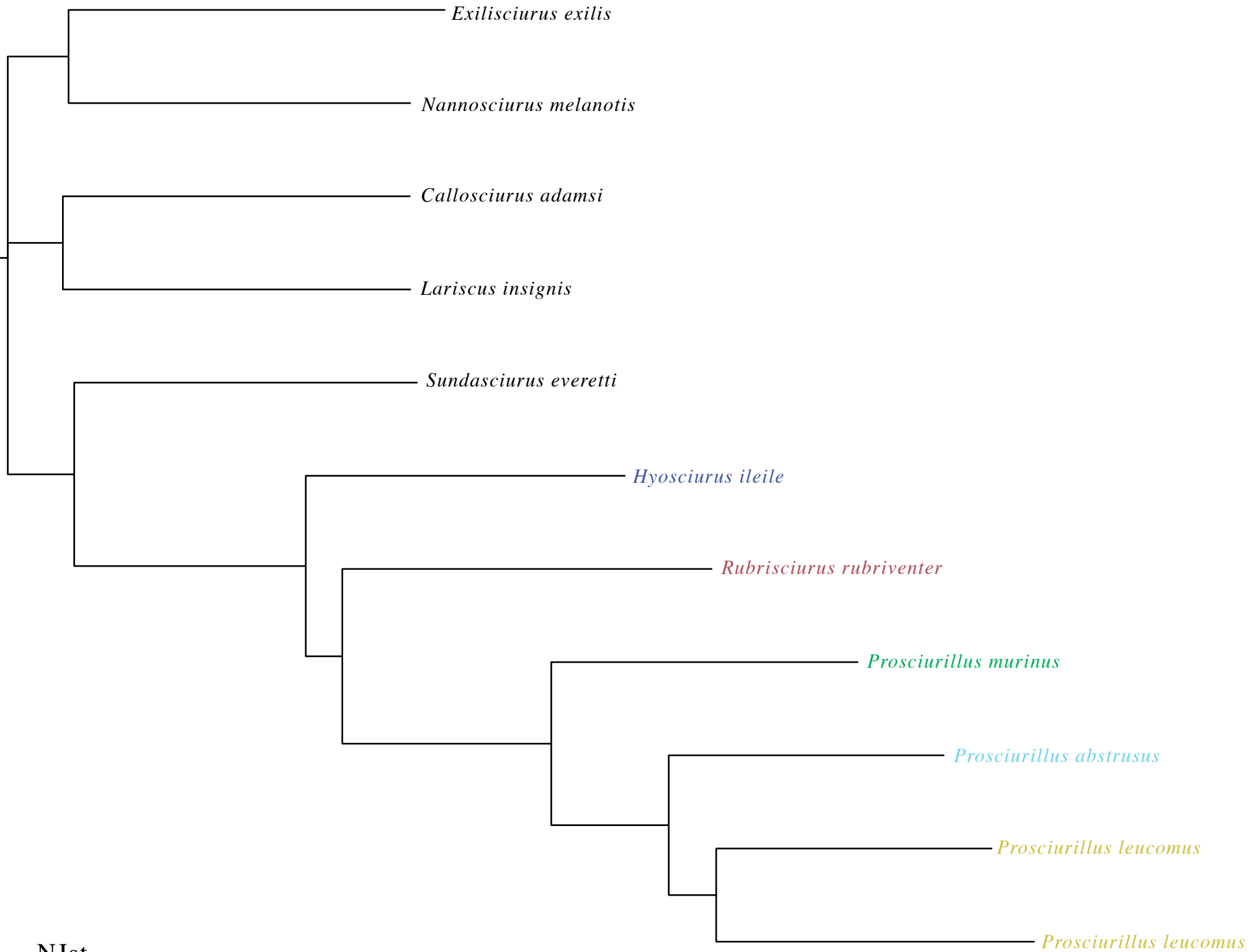

NJst

0.4

Supplement: Additional file 3: Figure S2. — Graphs showing information from two subsets of the incomplete matrix of UCE loci. The left column contains all loci enriched (4046 total) and the right column shows all loci which contain at least three informative sites (1137 loci). The top graph shows the overall length distribution across all loci for both subsets, the middle shows the number of informative sites, and the bottom shows the total number of differences across the loci. Note the two show very similar patterns, which was justification to use a smaller, yet equally informative subset of all loci. (PDF 194 kb) [file 12862_2016_650_MOESM3_ESM.pdf]
